# Corrosive Effect of Nifedipine in the Upper Gastrointestinal Tract

**DOI:** 10.1155/DTE.6.39

**Published:** 1999

**Authors:** Alexandra Lavy

**Affiliations:** Gastroenterology Department Rambam Medical Center Haifa Israel

## Abstract

Upper gastrointestinal tract mucosa is prone to injury. Drugs may disturb gastric mucosa protective mechanisms and cause damage. Injury by NSAIDs is a well described complication. Nifedipine, a widely used drug, was not described before as having a potential to damage gastrointestinal mucosa. We describe here, two patients, who developed esophageal and gastric mucosal damage, probably related to Nifedipine ingestion.


*Diagnostic and Therapeutic Endoscopy*, Vol. 6, pp. 39–41
Reprints available directly from the publisher
Photocopying permitted by license only

© 1999 OPA (Overseas Publishers Association) N.V.
Published by license under
the Harwood Academic Publishers imprint,
part of The Gordon and Breach Publishing Group.
Printed in Malaysia.

### Corrosive Effect of Nifedipine in the Upper Gastrointestinal Tract

ALEXANDRA LAVY*

*Gastroenterology Department, Rambam Medical Center, Haifa, Israel*

*(Received 3 August 1998; Revised 22 April 1999; In final form 14 June 1999)*

**Upper gastrointestinal tract mucosa is prone to injury. Drugs may disturb gastric mucosa protective mechanisms and cause damage. Injury by NSAIDs is a well described complication. Nifedipine, a widely used drug, was not described before as having a potential to damage gastrointestinal mucosa. We describe here, two patients, who developed esophageal and gastric mucosal damage, probably related to Nifedipine ingestion.**

*Keywords:* Esophagitis, Gastric ulcer, Nifedipine

Injury to the upper gastrointestinal tract due to drugs is not uncommon [1–4]. Gastritis caused by NSAIDs is a well described and feared complication [5–12]. Nifedipine was not recognized before as being potentially harmful to the stomach and esophagus. We present here two patients who suffered severe damage, probably due to the ingestion of Nifedipine.

### CASE NO. 1

A 62 year old male was admitted to the hospital after vomiting a large amount of blood. The man had hypertension and was taking propranolol, nifedipine and enalapril maleate regularly.

Upon admission he was pale, his blood pressure was 140/70 and his pulse rate was 80/min. Emergency gastroscopy revealed a deep fundic ulcer in which a pill of nifedipine was firmly embedded (Fig. 1). The pill was removed and the patient was treated with cimetidine and recovered.

### CASE NO. 2

A 67 year old man presented with dysphagia for solid food. The man had diabetes mellitus and hypertension and was treated with glibenclamide, enalapril maleate and nifedipine. Gastroscopy revealed severe damage to mid-esophagus (Fig. 2). Biopsies for *Candida albicans*, Herpes virus, and

* Tel.: 972-4-8542895. Fax: 972-4-8515710.

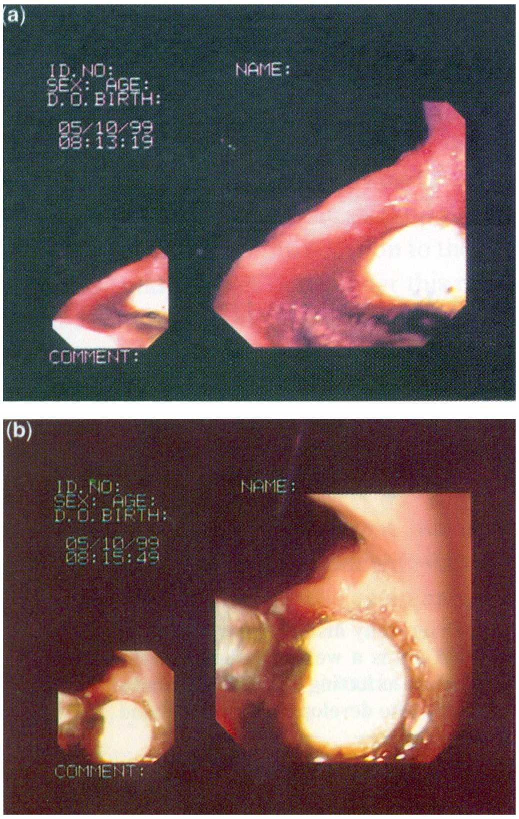

FIGURE 1 A pill embedded in an ulcer crater.

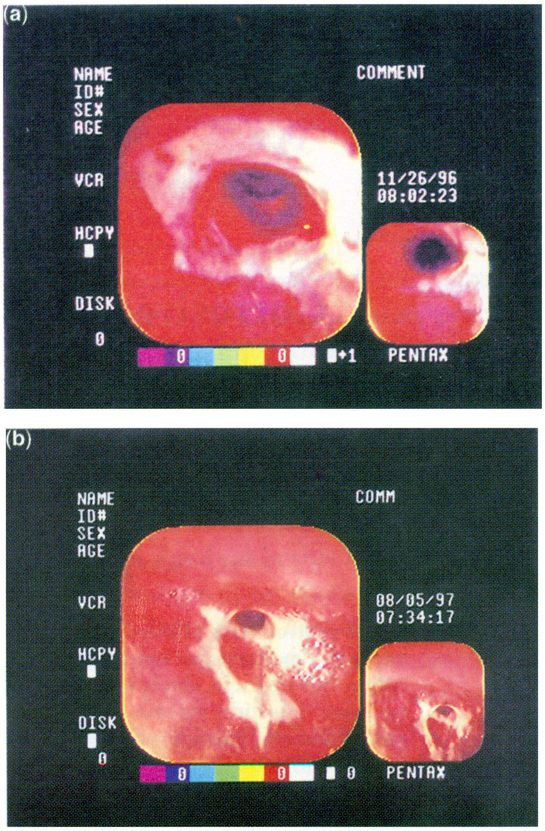

FIGURE 2 Short inflamed segment in mid-esophagus.

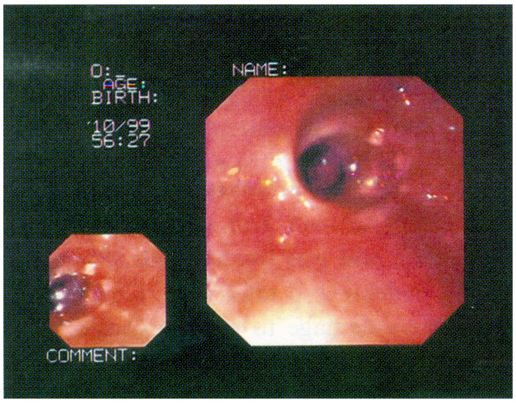

FIGURE 3 Marked improvement in patient no. 2.

tuberculosis were negative. He had extensive work-up for various inflammatory and infectious diseases, motility studies, pH 24 h monitoring and small bowel X-ray series. All tests were normal.

The patient did not respond neither to omeprazole, sucralfate nor to acyclovir treatment and needed repeated esophageal dilatations. At that time nifedipine was stopped. The patient gradually improved and the lesion disappeared (Fig. 3).

### DISCUSSION

Iatrogenic damage to the upper gastrointestinal tract is a well feared problem. The damage caused by NSAIDs is a world-wide problem and the wide use of aspirin adds to it. Corrosive damage to the upper gastrointestinal tract may cause pain, bleeding, and endanger the life of the patient.

The possible side effect of nifedipine causing severe mucosal damage, was not reported before. The harmful effect on esophageal mucosa was noted by others but not reported. In a single case report from Japan [13] severe esophagitis was reported in a woman, creating esophagogastric fistula. The authors noted that the woman began taking nifedipine one week earlier, but did not make the link. Due to the very common use of nifedipine, we suggest that caution should be taken whenever gastrointestinal symptoms appear.

### References

[1] Younossi, Z.M., Strum, W.B., Schatz, R.A. *et al.* Effect of combined anticoagulation and low-dose aspirin treatment on upper gastrointestinal bleeding. *Dig. Dis. Sci.* 1997; **42**: 79–82.

[2] Smalley, W.E., Griffin, M.R., Fought, R.L. *et al.* Excess costs from gastrointestinal disease associated with nonsteroidal anti-inflammatory drugs. *J. Gen. Intern. Med.* 1996; **11**: 461–469.

[3] Szabo, S. and Goldberg, I. Experimental pathogenesis: drugs and chemical lesions in the gastric mucosa. *Scand. J. Gastroenterol.* Suppl. 1990; **174**: 1–8.

[4] Elta, G., Behler, E., Nostrant, T. *et al.* Endoscopic diagnosis of gastritis: causative factors in 100 patients. *South Med. J.* 1987; **80**: 1087–1090.

[5] Oren, R., Ligumsky, M., Lysy, J. *et al.* Gastro-duodenal injury associated with intake of 100–325 mg aspirin daily. *Postgrad. Med. J.* 1993; **69**: 712–714.

[6] Zimmerman, J., Arnon, R., Ligumski, M. *et al.* Acute upper gastrointestinal bleeding in Jerusalem 1988–91: causes, characteristics and relation to nonsteroidal anti-inflammatory drugs. *Isr. J. Med. Sci.* 1993; **29**: 292–297.

[7] Mehta, S., Dasarathy, S., Tandon, R.K. *et al.* A prospective randomized study of the injurious effects of aspirin and naproxen on the gastroduodenal mucosa in patients with rheumatoid arthritis. *Am. J. Gastroenterol.* 1992; **87**: 996–1000.

[8] Bellary, S.V., Isaacs, P.E. and Lee, F.I. Upper gastrointestinal lesions in elderly patients presenting for endoscopy: relevance of NSAID usage. *Am. J. Gastroenterol.* 1991; **86**: 961–964.

[9] Misra, R., Pandey, H., Chandra, M. *et al.* Effects of commonly used NSAIDs on gastric mucosa. A clinico-endoscopic and histopathological study. *J. Assoc. Physicians India* 1990; **38**: 913–915.

[10] Yoshida, N., Yoshikawa, T., Nakamura, Y. *et al.* Role of neutrophil-mediated inflammation in aspirin induced gastric mucosal injury. *Dig. Dis. Sci.* 1995; **40**: 2300–2304.

[11] Stodolnik, E., Maurer, P., Hoigne, R. *et al.* Risk of acute upper gastrointestinal bleeding in patients with ulcerative disease and treatment with non-steroidal anti-inflammatory drugs (NSAIDs) – Results from the Comprehensive Hospital Drug Monitoring Berne (CHDM). *Eur. J. Clin. Pharmacol.* 1990; **38**: 31–35.

[12] Tenenbaum, J. Non-steroidal anti-inflammatory drugs (NSAIDs) cause gastrointestinal intolerance and major bleeding – or do they? *Clin. Invest. Med.* 1987; **10**: 246–250.

[13] Hoshino, E., Umeda, N., Matsueda, K. *et al.* Double cardia. An unusual sequela of reflux esophagitis with ulcer. *Dig. Dis. Sci.* 1990; **35**: 638–640.

